# Unintended drug exposure during pregnancy in clinical trials – a survey in early drug development 

**DOI:** 10.5414/CP203788

**Published:** 2020-11-03

**Authors:** Katharina Erb-Zohar, Hildegard Sourgens, Kerstin Breithaupt-Groegler, Christine Klipping

**Affiliations:** Association for Applied Human Pharmacology (AGAH e.V.), Hamburg, Germany

**Keywords:** unintended pregnancy, clinical trial, drug exposure via parent, early drug development, survey

## Abstract

Purpose: To collect information on unintended drug exposure during pregnancy in early clinical drug development. Materials and methods: Questionnaire mailed in autumn 2015 to members of human pharmacology societies in Europe for anonymous responses via the online tool SurveyMonkey. Results: 53 of the ~ 700 addressees participated in the survey. 23 female trial participants and 11 female partners of male trial participants were exposed to investigational medicinal products during unintended pregnancies in a clinical trial. Most survey respondents confirmed adequate contraceptive methods by in/exclusion criteria and the use of pregnancy tests in female trial participants at screening and before the first dose. The last menstrual period was documented less frequently (at screening: 28 of 44, before first dose: 5 of 44 respondents). A considerable proportion of respondents denied the routine use of compliance checks about the appropriate use of contraceptive methods, had no procedures in place if contraceptive methods failed, and did not train physicians in instructing trial participants about the appropriate use of contraceptive methods. Conclusion: The methods to avoid unintended pregnancies during participation in a clinical trial need improvement and should include (i) pregnancy tests, (ii) documentation of last menstrual period before the first dose, (iii) compliance checks of the appropriate use of contraceptive methods, and (iv) training of trial physicians. Procedures should be in place for what to do if contraceptive methods fail.

**What is known about this subject**

- Pregnancies in early clinical drug development must be avoided
- Appropriate methods to avoid unintended pregnancies are to be defined in the clinical trial protocol
- Guidance is provided in the ICH M3(R2) and the respective European Clinical Trial Facilitation Group recommendations (CTFG 2014)

**What this study adds**

- This AGAH survey revealed that unintended pregnancies occur in early clinical drug development
- Regulatory recommendations are not fully implemented
- The methods to avoid unintended pregnancies during participation in a clinical trial need improvement

## Introduction 

A series of workshops related to early-phase clinical development has been hosted by the scientific society “Association for Applied Human Pharmacology” (AGAH e.V.) in Germany since 2012. In 2015, members from the pharmaceutical industry, contract research organizations, academia, ethics committees, and the German competent authority discussed unintended exposure to an investigational medicinal product (IMP) during pregnancy in clinical trials and its prevention [1]. 

In early drug development, data on reproduction toxicity are in general not yet available. According to the ICH M3(R2) Guidance [2]:

- “Men can be included in Phase I and II trials before the conduct of the male fertility study since an evaluation of the male reproductive organs is performed in the repeated-dose toxicity studies”
- “For women of childbearing potential (WOCBP) there is a high level of concern for the unintentional exposure of an embryo or foetus before information is available concerning the potential benefits versus potential risks”

If reproduction toxicity studies are not available, and WOCBP are planned to be included in a clinical trial, the guideline recommends limiting the risk by taking precautions to prevent pregnancy by (i) pregnancy testing (e.g., based on the β-subunit of HCG), (ii) use of highly effective methods of birth control, and (iii) trial entry only after a confirmed menstrual period. 

To enable trial participation of WOCBP, the ICH M3(R2) Guidance [2] further recommends:

- Informed consent should be based on known risks to the embryo/fetus related to reproduction toxicity. A general assessment of potential toxicity of pharmaceuticals with related structures or pharmacological effects should be provided
- If no relevant reproductive information is available, the potential for unidentified risks to the embryo or fetus should be communicated as well
- Subjects should be instructed to use adequate contraceptive measures during the period of drug exposure which could exceed the length of the trial
- Pregnancy tests are required during the trial to ensure compliance with the contraceptive measures planned in the trial protocol

In Europe, the Clinical Trials Facilitation Group (CTFG) has issued further recommendations related to contraception and pregnancy testing in clinical trials [3]. They are intended to supplement “existing guidelines related to embryofetal risk mitigation and to provide practical guidance on contraception use and pregnancy testing in clinical trials”. 

A survey was conducted (i) to collect information on unintended pregnancies during clinical trials in early drug development and (ii) to learn from current experience whether applied precautionary measures are in line with current guidances [2, 3] and appropriate. 

## Materials and methods 

Definitions used are:

- Drug exposure via parent (DEVP)
- Unintended drug exposure during pregnancy in a clinical trial

The survey addressed specialists working in early-phase drug development in Germany, Belgium, France, and the United Kingdom. 

The questionnaire (Online supplemental material) comprised 13 questions for anonymous responses by means of the online tool SurveyMonkey. Addressees in Germany were AGAH members, delegates of the VfA (“Verband forschender Arzneimittelhersteller”), BPI (“Bundesverband der pharmazeutischen Industrie”), BAH (Bundesverband der Arzneimittel-Hersteller), DGPharMed (“Deutsche Gesellschaft für Pharmazeutische Medizin”); in Belgium members of the Belgian Association of Phase I units (BAPU); in France members of Club Phase I; and in the United Kingdom members of the Association for Human Pharmacology in the Pharmaceutical Industry (AHPPI). Electronic mailings were on November 6, 2015 and November 10, 2015. The survey was closed on November 16, 2015. 

The questions covered the following topics (see questions (Q) provided in Online supplemental material):

- Characteristics of survey participants (Q1, Q2)
- Number and cause of unintended pregnancies in female participants and female partners of male trial participants in clinical trials combined in one protocol (Q3 – Q8)
- Age of women becoming accidentally pregnant in a clinical trial (Q9)
- Preventive measures (Q10 – Q13)

It cannot be excluded that different participants belonging to the same organization referred to the same trial protocol(s). In consequence, a qualitative rather than quantitative interpretation of results is indicated. Not all survey participants answered all questions. For the respective questions, the total number of responses is given (Table 1). 

## Results 

### Survey participants 

A total of 53 of ~ 700 addressees answered the questionnaire. The majority came from contract research organizations (19), the pharmaceutical industry (15), but also from academia (8), independent consultants (8), biotechnology (2), or regulatory authority (1). 

Participants were from Germany (47), The Netherlands (3), Belgium (1), Luxembourg (1), and South Africa (1). 

### Number of unintended pregnancies in female trial participants (48 respondents) 

15 survey participants reported that 23 female clinical trial participants were unintentionally exposed to IMP during pregnancy in a clinical trial they sponsored or conducted during the past 10 years. 33 survey participants denied the occurrence of such pregnancies. 

### Number of unintended pregnancies in female partners of male trial participants (46 respondents) 

A total of 10 survey participants reported that 11 female partners of male clinical trial participants were unintentionally exposed to IMP during pregnancy in a clinical trial they sponsored or conducted during the past 10 years. 36 survey participants denied the occurrence of such pregnancies. 

### Main causes of unintended pregnancies (10 respondents, multiple entries possible) 

These included (i) non-compliance with trial-specific contraceptive methods (9), (ii)  failure of contraceptive methods (3), and (iii) failure of the pregnancy test to detect pregnancy at screening or before first dose (1). 

### Measures implemented in early-development clinical trials to mitigate the risk of unintended pregnancies 

Table 1 gives an overview. Most survey respondents confirmed adequate contraceptive methods by in/exclusion criteria and the use of pregnancy tests in female trial participants at screening and before the first dose. The last menstrual period was documented less frequently. A considerable proportion of respondents denied the routine use of compliance checks about the appropriate use of contraceptive methods, had no procedures in place if contraceptive methods failed, and did not train physicians in instructing trial participants about the appropriate use of contraceptive methods. 

## Discussion 

The main outcome of this AGAH survey demonstrates that the occurrence of unintended pregnancies is a relevant issue in early drug development despite considerable effort to avoid them. Taking into account that 53 out of ~ 700 addressees responded, it may be assumed that the unintended exposure to IMPs in early pregnancy is higher than reported here. 

### Limitations of the reported survey 

The number of participants is not a representative sample of all parties conducting clinical trials in Europe. Participants from the same organization may have referred to the same trials. 

### Strengths of the reported survey 

To provide (i) data with respect to a topic with considerable lack of information, (ii) data on failure of/or compliance with contraceptive methods, (iii) data on training of physicians in instructing trial participants how to use the contraceptive methods as specified in the clinical trial protocol. 

Despite the advice provided in the ICH M3(R2) guidance [2] and the Clinical Trial Facilitation Group (CTFG) recommendations [3], unintended pregnancies in biomedical research are still a matter of concern as evidenced in recent publications [4, 5, 6, 7]. A 2018 paper [6] reports wide variation in pregnancy testing plans in clinical trials, leading to the potential for inadequate protection against embryonic or fetal exposure in some cases and unnecessary burdens on research participants in others. The authors recommend that study protocols should clearly address the rationale for pregnancy testing and the handling of positive and indeterminate tests. An industry survey on birth control in clinical trials revealed that few companies collected data in a manner that would allow retrospective understanding of the reasons for the failure of birth control in clinical trials [7]. Among other causes, one reason for unintended pregnancies in the setting of clinical trials could be that the perspectives of women themselves on contraception in research are largely unexplored [4]. 

If contraceptive methods fail in a clinical trial, the following procedures are useful in identifying the risk of having become or becoming pregnant and to estimate the usefulness of emergency contraception:

- Identification of exact kind and time point of contraceptive failure (i.e., rupture of condom, forgotten pill, etc.)
- Cycle history (identification of date of last menstruation or withdrawal bleeding)
- Urine pregnancy test
- If possible, transvaginal ultrasound for determination of ovarian activity
- Blood sampling for determination of beta-human choriogonadotropin (β-HCG), estradiol, luteinizing hormone (LH), and progesterone

There was agreement among participants of the workshop that good practices to avoid unintended pregnancies in early drug development comprise:

- Implementation of adequate contraceptive methods in the clinical trial protocol
- Pregnancy tests in female trial participants depending on the kinetics of the tested compound. At least screening before 1^st^ dose, at appropriate time points thereafter, and upon completion of the clinical trial
- Written subject information concerning the adequate contraceptive methods to be used during the trial and the necessity avoiding DEVP as well as measures to be taken in case of possibly failed contraception before or during the trial
- Training of trial physicians on how to instruct trial participants about the appropriate use of adequate contraceptive methods as well as measures to be taken in case of possibly failed contraception (i.e., forgotten use of contraceptive measure or ruptured condom)
- Routine compliance checks whether enrolled subjects are appropriately using the contraceptive methods as per protocol (e.g., intake of the oral contraceptive at the trial site in case of high risk/diaries for the documentation of the use of contraceptive measures)
- Standard procedures in place regarding what to do if contraceptive methods failed (e.g., emergency contraception)
- Decision on further measures to avoid unnecessary elective pregnancy terminations
- Compliance with measures indicated by the CTFG publication [3]

## Conclusion 

Female clinical trial participants and female partners of male clinical trial participants must not become pregnant during early drug development; however, this AGAH survey shows that pregnancies did occur. According to the discussions during the workshop, the methods to mitigate the risk of unintended pregnancies need further improvement and should include (i) written information of the trial participants regarding contraceptive measures and the necessity of avoiding drug exposure via parent, (ii) repeated pregnancy tests, (iii) documentation of last menstrual period before the first dose, (iv) compliance checks of the appropriate use of contraceptive methods, (v) training of trial physicians. Procedures should be in place for what to do if contraceptive measures fail. Lessons learned from unintended pregnancies should result in appropriate modification of hitherto implemented procedures. This will mitigate the risk of DEVP in early-phase clinical trials. 

## Acknowledgment 

We thank Sam Welgemoed for her technical support (Richmond Pharmacology, London and St George’s University London, United Kingdom). We thank all participants of the survey for their contributions. 

## Funding 

Survey: None. 

Open access provided by AGAH e.V. 

## Conflict of interest 

None. 

**Table 1.** Measures implemented in early-development clinical trials to mitigate the risk of unintended pregnancies.

Topic	Responses
Methods routinely used to avoid unintended drug exposure during pregnancy in clinical trials (44 respondents)	Adequate contraceptive methods in in/exclusion criteria: 43 In female trial participants, pregnancy test at screening: 39 In female trial participants, pregnancy test before first dose: 37 In female trial participants, documentation of last menstrual period (LMP) at screening: 18 In female trial participants, documentation of LMP before first dose: 5
Routine compliance checks whether subjects used contraceptive methods, how and when they used contraceptive methods (42 respondents)	Yes: 23 No: 19
Procedures in place on what to do if contraceptive methods failed (42 respondents)	Yes: 25 No: 17
Training physicians in instructing trial participants on how to use the contraceptive methods as specified in the clinical trial protocol (42 respondents)	Yes: 26 No: 16

## Supplemental material

Supplemental material Appendix 1
