# Supplementary material for: Unintended drug exposure during pregnancy in clinical trials – a survey in early drug development
Source: Int J Clin Pharmacol Ther. 2020 Nov 3;59(1):26–30. doi: 10.5414/CP203788 (PMC7737523; doi:10.5414/CP203788)
Supplement: Supplemental material [file intjclinpharmacol-59-026-S01.pdf]

Online Supplemental Material

Appendix 1

Q: Question, below each questions the options that could be selected as answer are given

Q1 Please select from the list below the option that best describes you or your affiliation
Pharmaceutical industry, Biotech, CRO, University, Consultant, Other

Q2 In which country are you based?
Belgium, France, Germany, United Kingdom, Other

Q3 Has any female clinical trial participant been unintendedly exposed to drug during pregnancy in a clinical trial you sponsored or conducted?
Yes, No

Q4 If yes, how many how many during the past 10 years?
Number of unintended pregnancies in a clinical trial with drug exposure via mother: __

Q5 Has any female partner of a male clinical trial participant exposed to drug become unintendedly pregnant during a trial you sponsored or conducted? *Drug exposure of a male trial participant before/around the time of conception and/or during pregnancy of his female partner may have an effect on the offspring by a drug effect on the spermatozoon or by the presence of the drug in the seminal fluid.*
Yes, No

Q6 If yes, how many during the past 10 years?
Number of unintended pregnancies in a clinical trial with drug exposure via father: __

Q7 Why did this/these unintended drug exposure/s via parent during pregnancy happen?
Options (multiple answers possible): Failure of contraceptive methods; specify method that failed_____; Non-compliance with study-specific contraceptive methods; Inadequate contraceptive methods in IN/EXclusion criteria; Failure of pregnancy test to detect pregnancy at screening or before the first dose; Other; specify:_____

Q8 How old were the women becoming unintendedly pregnant in a clinical trial you sponsored or conducted?
Options (multiple answers possible): 18-25 years / 25-30 years / 30-40 years / >40 years / unknown

Q9 Which methods do you routinely use to avoid unintended drug exposure during pregnancy in clinical trials?
Options (multiple answers possible): Adequate contraceptive methods in IN/EXclusion criteria; In female trial participants pregnancy test at screening; In female trial participants pregnancy test before first dose; In female trial participants documentation of last menstrual period

Q10 If yes when?
At screening / before first dosing / after last dosing / other timing; specify:_____If yes, please state the issues

Q11 Are you doing routinely compliance checks, e.g. asking the subjects whether, how and when they used contraceptive methods?
Yes, No

Q12 Do you have procedures in place what to do if contraceptive methods failed?
Yes, No

Q13 Do you train physicians in instructing trial participants how to use the contraceptive methods as specified in the clinical trial protocol?
Yes, No
